# The expression of syndecan-1, syndecan-4 and decorin in healthy human breast tissue during the menstrual cycle

**DOI:** 10.1186/1477-7827-8-35

**Published:** 2010-04-16

**Authors:** Gunilla Hallberg, Eva Andersson, Tord Naessén, Gunvor Ekman Ordeberg

**Affiliations:** 1 Department of Women's and Children's Health, Uppsala University, S-751 85 Uppsala, Sweden; 2 Department of Women's and Children's Health, Karolinska Institute, Solna S-171 76 Stockholm, Sweden

## Abstract

**Background:**

In order to unravel the interactions between the epithelium and the extra cellular matrix (ECM) in breast tissue progressing to cancer, it is necessary to understand the relevant interactions in healthy tissue under normal physiologic settings. Proteoglycans in the ECM play an important role in the signaling between the different tissue compartments. The proteoglycan decorin is abundant in the breast stroma. Decreased expression in breast cancer tissue is a sign of a poor tumor prognosis. The heparane sulphate proteoglycans syndecan-1 and syndecan-4 promote the integration of cellular adhesion and proliferation. The aim of this study was to investigate the gene expression and location of decorin, syndecan-1 and syndecan-4 in the healthy breast during the menstrual cycle.

**Methods:**

Tissue from healthy women undergoing breast reduction plastic surgery was examined using immunohistochemistry (n = 38) and Real-Time RT-PCR (n = 20). Both parous and nulliparous women were eligible and the mean age of the women was 34(+/- 10 years) with regular menstrual cycles (28 +/- 7 days). None of the women had used hormonal treatment the last three months. The women were randomized to needle biopsy two months before the operation in the follicular or luteal menstrual phase and for another biopsy at the operation in the opposite phase. Serum samples were obtained to characterize the menstrual phase. The Wilcoxon signed rank test and Mann Whitney test were used for statistical analyses.

**Results:**

By real time-RT-PCR the gene signal for all three proteoglycans; decorin (p = 0.02) and syndecan-1 (p = 0.03) and syndecan-4 (p = 0.02) was significantly lower among parous women in the luteal phase than in the follicular phase. Immunohistochemistry confirmed the identification of the proteins but no significant difference between menstrual phases was observed. Serum samples verified the menstrual phase.

**Conclusions:**

Our study shows, for the first time in the healthy breast, a significantly lower expression of the genes for the three proteoglycans, decorin, syndecan-1 and syndecan-4 in the luteal phase during the menstrual cycle. These changes were registered under normal physiologic conditions. Since ECM molecules appear to be involved in tumor progression, these findings in the normal breast could constitute a base for further studies in women receiving hormonal therapy or those with breast cancer.

## Background

During the menstrual cycle, the human mammary gland undergoes histologic changes in both the epithelial and stromal compartments. *In vitro *studies have implied that the extracellular matrix (ECM) plays a role in modulating proliferation, differentiation and gene expression [1]. Increased synthesis and degradation of the ECM, components seems to be associated with breast cancer and its prognosis [2,3]. Proteoglycans, which are strategically located on the surface of the ECM cells and in the pericellular matrix, have an important role in stromal-epithelial interactions and signaling and are able to control the activity of extracellular regulatory proteins [4]. In young premenopausal women, 15% of breast tissue consists of epithelium: this is gradually replaced by fat tissue later in life. Among postmenopausal women, only 5% of the breast contains epithelium [5] while breast stroma composed of collagen, fibroblasts, endothelial cells, adipocytes and a macromolecular network of proteoglycans accounts for more than 80% of the breast volume [6].

Several authors have described the pattern of the proteoglycan expression in the light of breast cancer progression [7,8]. The expression of decorin, which is abundant in the stroma, can for example, indicate the prognosis of a tumor [9] and decorin can also diminish tumor growth in rat experiments [10]. The syndecans are heparan sulphate proteoglycans (HSPG) that are expressed in the basement membrane and the epithelial cells. Among postmenopausal women with breast cancer or dense-mammographic breast tissue, the expression of these proteoglycans changes from the epithelium to the stroma [11,12].

Only a few workers have addressed the role of the proteoglycans in the stroma under normal physiologic conditions in the healthy breast [13]. Various hormonally regulated epithelial and stromal events, possibly associated with changes in the extracellular matrix, occur during the menstrual cycle. Knowledge of the normal expression of gene signals for these proteoglycans during the menstrual cycle is therefore of importance. The purpose of this study was to examine the location and gene expression of the proteoglycans decorin, syndecan-1 and syndecan-4 in healthy breast tissue, with particular attention to the ECM, during the follicular and luteal phases of the menstrual cycle.

## Methods

### Subjects

Women on the waiting list for mammoplasty surgery for breast reduction at the Department of Plastic Surgery, University Hospital, Uppsala, Sweden were recruited for this study. Healthy non-pregnant premenopausal women were eligible if they had regular menstrual periods (28 ± 7 days) and had not used hormonal contraception during the last three months. Nulliparous (n = 15) or parous (n = 23) women were included. The study was approved by the Ethics Committee of the Faculty of Medicine, Uppsala University, Uppsala, Sweden.

### Study design

The participants were randomized to breast reduction surgery timed according to their menstrual cycle phase (follicular or luteal). A preoperative mammography and a needle biopsy, preferably in the right breast, were performed during the alternate menstrual phase, two months before the operation and at the operation in the opposite phase. Serum samples were collected on the same day to confirm the assumed phase. On the operation day, breast tissue and a second serum sample were collected. The reduction mammoplasty surgery was performed according to routine procedures.

### Sampling of breast tissue

For all women the samples were taken two months before and at the operation. Samples were formalin-fixed, as well as frozen in liquid nitrogen at both occasions. Two months before the operation a needle biopsy was taken. The biopsy was taken from the right breast using an automatic double spring gun "Manan" pro-Mag 2.2 with a 22 mm stroke lens and a 14 Gauge needle (2.1 mm: ×16 cm with a 17 mm notch U. S. Biopsy division of Promix Inc USA). Immediately after the main surgery, parenchymal tissue was dissected from the adipose tissue, frozen in liquid nitrogen and stored at -70° until analyzed. At the same time parenchymal tissue was fixed in 4% paraformaldehyde and later paraffin-embedded. The paraffin-embedded tissue was sectioned and mounted on Fisher slides. The slides were stored in a dark room at a cool temperature.

The immunohistochemistry analysis was carried out on biopsies from 15 nulliparous and 23 parous women. RNA-preparations containing sufficient material for real-time reverse-transcription polymerase chain reaction (RT-PCR) analyses were available from 20 women.

### Immunohistochemistry

Formalin-fixed tissue taken two months before the operation and at the reduction mammoplasty was paraffin-embedded. The paraffin-embedded tissue was sectioned and mounted on Fisher slides. The slides with paraffin-embedded sections were preheated for 30 minutes at 60° in an oven. The slides were then heated with DIVA DECLOAKER (Biocare Medical, Concord, CA) in a 2100-RETRIEVER (Histolab, Gothenburg, Sweden) for 20 minutes for antigen retrieval, after which the tissue sections were rinsed in HOT RINSE (Biocare Medical) to guarantee complete deparaffinization. After rinsing in Tris-buffered saline (TBS) (Biocare Medical), endogenous peroxidaze activity in all sections was quenched by incubation in PEROXIDAZED (Biocare Medical) for 5 minutes in darkness at room temperature. After incubation with BACKGROUND SNIPER (Biocare Medical) for 10 minutes, the sections were incubated with the primary monoclonal antibodies decorin (conc. 1 mg/ml, Code No 270425, Clone 6-B-6, 1:3000; Seikagaku, Tokyo, Japan) and syndecan-1, (Code No MCA681, Clone No B-B4 1:400; Serotec, Oxford, England) and syndecan-4 (conc. 200 μgIgG/ml, Code No Sc-9499, 1:800; Santa Cruz, CA, USA) for 60 minutes (Table 1). Subsequently, the biotin-free detection system mouse-probe HRP polymer kit MACH 3™ (Biocare Medical, Walnut Creek, CA) was used for decorin and syndecan-1, while the Goat-HRP polymer kit MACH 3™ was used for syndecan-4. The reaction was developed using the diaminobenzidine DAB kit (Biocare Medical, Walnut Creek, CA). The TBS rinse was used between each step. The sections were then counterstained with hematoxylin. Finally, the slides were mounted with Pertex^® ^(Histolab products AB, Gothenburg, Sweden). In each assay, controls for specificity were carried out using primary antibodies preincubated with blocking peptides for syndecan-4 (Santa Cruz, Biotechnology) and with mouse IgG1 negative control for syndecan-1 and decorin. In all cases, immunoreactivity in the breast biopsy was classified blind by two independent observers using conventional light microscopy (GH, EA). The staining intensity was graded on a scale of (0) = no staining, (1) = faint staining, (2) = moderate staining and (3) = strong staining.

**Table 1:** Assay ID used in real-time RT-PCR and antibodies used in Immunohistochemistry

	Real Time PCR		Antibodies		
	**Assay ID**	**Reference Sequence database accession number**	**No, manufacturer**	**Type**	**Dilution**

Syndecan-1	Hs 00174579_m1	NM_001006946.1	MCA 681, Serotec, Oxford, England	Mouse monoclonal	1:400

Syndecan-4	Hs 00161617_m1	NM_002999.2	Sc-9499, Santa Cruz, CA, USA	Goat polyclonal	1:800

Decorin	Hs 00370385_m1	NM_133503.2	270425, Seikagaku, Tokyo, Japan	Mouse monclonal	1:3000

### Sample procedure for Real-Time RT-PCR

#### Tissue homogenization and extraction of RNA

The biopsies were homogenized frozen using a dismembranation apparatus (Retsch KG, Haan, Germany) to achieve a fine powder from which the total RNA was extracted using a Trizol reagent (Invitrogen, Carlsbad, CA, USA) according to the manufacturer's instructions.

#### Reverse Transcription (RT)

The concentration of total RNA was determined by Nanodrop™ 1000, only samples exhibiting an OD_260_/OD_280 _ratio higher than 1,8 were used and the quality of the total RNA was verified by running an agarose gel. The RT reaction used on 1 μg total RNA 1 μl (200 ng) of pd (N)_6_. Random Hexamer 5'-Phosphate primers (Amersham Biosciences, Pistacaway, NJ, USA), 1 μl of 10 mM dNTP (Amersham Biosciences) and sterile water added to 12 μl. The mixture was incubated for 5 min at 65°C, cooled and centrifuged. The reaction mixture consisting of 4 μl of 5 × First-Strand Buffer, 2 μl of 0.1 M DTT (Invitrogen, Carlsbad, California) and 1 μl (40 U/μl) Protector Rnase Inhibitor (Roche, Mannheim, Germany), was added and the resulting mixture was incubated for 2 min at 25°C. 1 μl (200 U/μl) of Superscript™ Rnase H^- ^Reverse Transcriptase (Invitrogen, Carlsbad, California, USA) was added to each tube and mixed and incubated in 10 minutes at 25°C. The RT step was carried out at 42°C for 50 min, followed by heating at 70°C for 15 min to inactivate the enzyme. The cDNA was stored at -70°C until used.

#### Real-Time PCR

The levels of mRNA encoding decorin, syndecan-1, and syndecan-4 were quantified using the Applied Biosystems 7300 Real-Time PCR system (Applied Biosystems, Foster city, CA, USA). All determinations were performed in triplicate in the Taqman Universal PCR master Mix (Applied Biosystems, Foster city, CA, USA) on 96-well optical PCR plates. Appropriate primers and probes were obtained from commercially available Taqman^® ^gene expression assays (Applied Biosystems). Assay IDs and Reference Sequence database accession numbers are presented in Table 1. Ribosomal 18 S RNA (Assay ID 4319413E, Applied Biosystems), hormone-stable and expressing ribosomal RNA, was used as a house-keeping gene. Each reaction used 5 μl diluted cDNA corresponding to 10 ng total RNA in 12.5 μl Universal Master Mix, 1.25 μl assay mixture and 6.25 μl sterile water. The real-time PCR reaction was carried out according to the manufacturer's protocol. The threshold cycles (C_T_), at which an increase in reporter fluorescence above the baseline signal was first detected, were determined. 18S was used as endogenous control and for normalization (ΔC_T_)of the mRNA levels for the gene of interest. The endogenous control was subtracted from respective gene giving the ΔC_T _as a reflection of the relative mRNA expression. Mean value of (ΔC_T_) for women in the follicular and luteal phase were calculated using the Mann Whitney U test. To compare the women in the follicular and luteal phase, the calculations of relative gene expression were done using the ΔΔC_T _method, where the women in the follicular menstrual phase were used as a control group. Because the amount of product doubles in each cycle the relative gene expression was calculated using the formula 2^-ΔΔCT^, given in the manufacturer's instructions. Serial dilutions were performed to measure cDNA with the housekeeping gene 18S on all biopsies to validate the exponential amplification previous to the Real-Time PCR. Serial dilutions of breast tissue cDNA made from purchased total RNA (Ambion, Austin, TX, USA) were used for validation of the experiment.

### Serum analysis

Commercial kits were used for analyzing the serum levels of estradiol and progesterone (Amerlite^®^, Johnson & Johnson clinical diagnostics Ltd, Amersham, UK), and sex hormonebinding globulin (SHBG: Delfia, Wallac Oy, Turku, Finland). The analyses were performed according to the manufacturer's manual.

### Statistical analysis

The matched pair Wilcoxon signed rank test was employed to compare the paired immunohistochemistry results. Comparisons between the two independent groups in the real-time RT PCR test were carried out using the Mann-Whitney U test.

## Results

**Figure 1:**
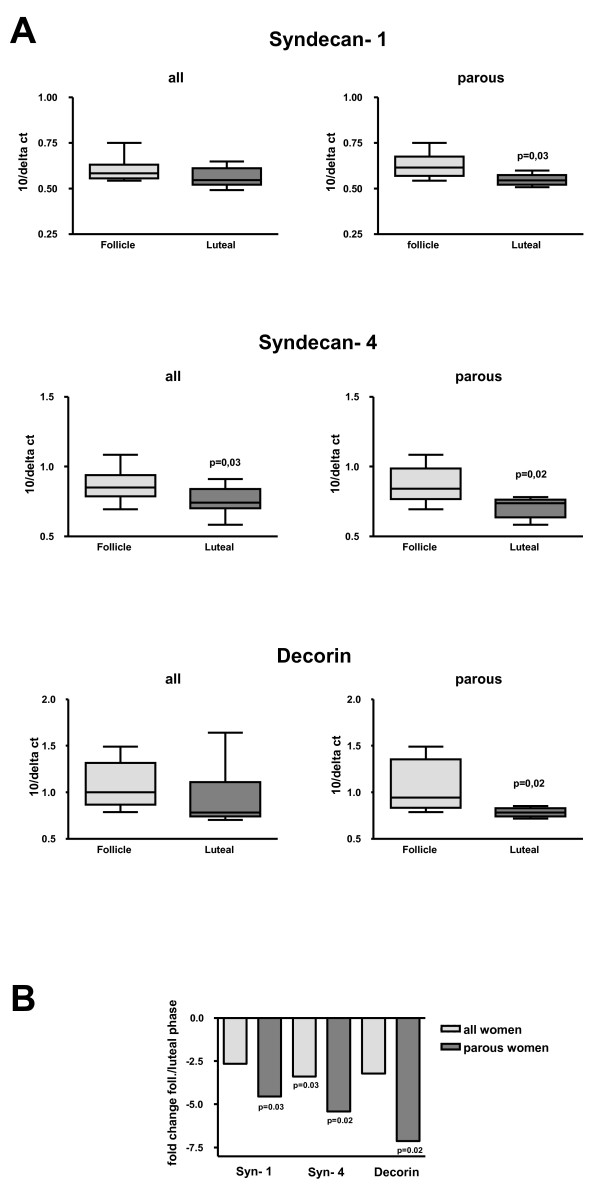
**A) The mean values of the ΔC_T _for women in the follicular and luteal phase in syndecan-1, syndecan-4 and decorin**. 18 S was used as an endogenous control and was used for normalization of the mRNA levels for the gene of interest. The endogenous control was subtracted from respective gene giving the ΔC_T _as a reflection of the relative mRNA. Since a higher ΔC_T _corresponds to a lower mRNA expression the ΔC_T _values are presented inverted as 10/ΔC_T_. **B) The mRNA expression of the proteoglycans syndecan-1, syndecan-4 and decorin in human breast tissue from 20 women, including 14 parous women.** The value from the follicular phase was used as control, using the ΔΔC_T _method.

**Figure 2:**
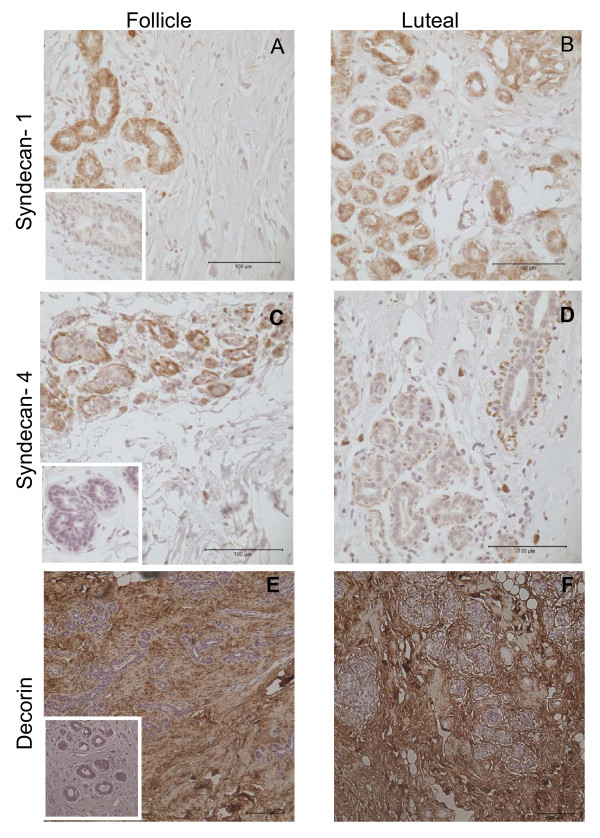
**Immunohistochemical staining of syndecan-1 (a, b), syndecan-4 (c, d) and decorin (e, f) in the follicular and luteal phases of the menstrual cycle**. The magnification was ×400 for syndecan-1 and syndecan-4 and × 200 for decorin. Negative controls were inserted in the follicular pictures a), c) and e). The matched pair Wilcoxon signed rank test was used to compare the immunohistochemistry results.

**Figure 3:**
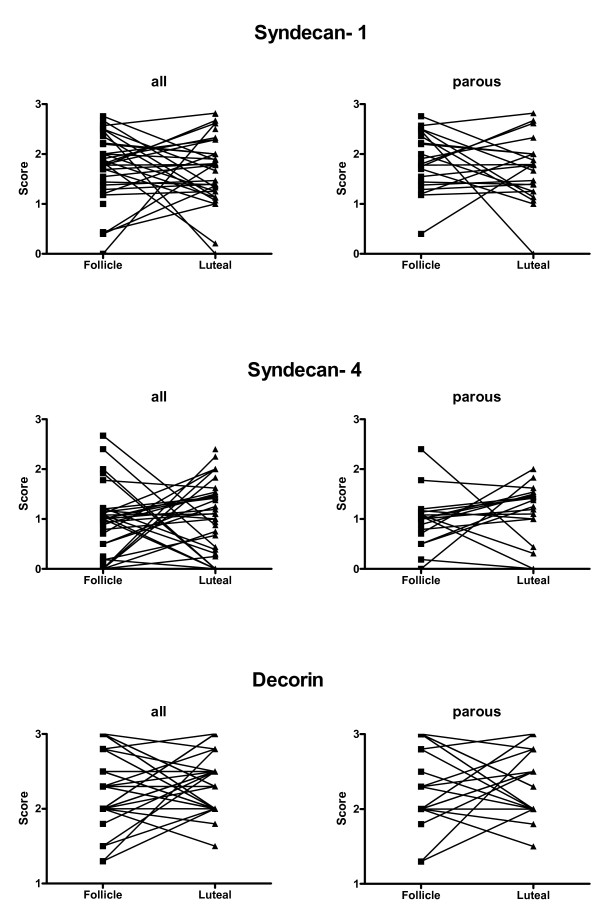
**Quantification of pair-matched immunohistochemical staining of syndecan-1, syndecan-4 and decorin in the follicular and luteal menstrual phase**. Semiquantitative scale from 0 to +++ was used.

Women in the study had a mean age of 34(+/- 10) years and a normal age of menarche 12.6 (+/- 1.5) years. The women had a BMI of 26.5(+/- 5) kg/m^2 ^and the serum level of estradiol and progesterone verified the corresponding menstrual phase (Table 2). Gene expression was calculated by ΔC_T_, normalized by the endogenous control 18S and using the mean value for the follicular and luteal phase. To compare the menstrual phases, the women in the follicular phase were a control group giving the magnitude of the fold decrease. Gene expression of syndecan-4 was significantly lower in the luteal phase than in the follicular phase of the menstrual cycle in the total group with a 2.6 fold decrease in expression (p = 0.03) and in parous women a more than five times lower expression (p = 0.02) in the luteal phase compared to the follicular (figure 1a and 1b). This was accompanied by an almost comparative five-fold lower gene expression for syndecan-1 in the luteal phase in parous women (p = 0.03). Gene expression of the small leucine-rich proteoglycan (SLRP) decorin was more than seven times lower in the luteal phase (p = 0.02) in parous women compared to the follicular phase(figure 1a and 1b). Although the same pattern of expression was seen in nulliparous women (n = 6) the difference did not reach statistical significance. This was probably due to low sample size. All proteins were well visualized by immunhistochemistry (figure 2). The staining of decorin in the ECM was strong but there was no difference in the staining intensity in the follicular as well as in the luteal phase. Stained syndecan-1 and -4 was clearly identified in the epithelial and the basement membranes. There were no significant differences in the distribution of syndecan-1 between the menstrual cycle phases. There was a prominent difference between patients rather than within each patient (figure 3). The inter-observer variations were for syndecan-1, syndecan-4 and decorin; rs = 0.82, rs = 0.91 and rs = 0.48; p < 0.0001 for all. All control sections were negative, as shown in Figures 2 a), c) and 2 e).

**Table 2:** Clinical characteristics of women included in the study

	Age Year	BMI (kg/m^2^)	Menarche (age)	P_4 _foll. (nmol/l)	P_4 _lut. (nmol/l)	E_2 _foll (pmol/l)	E_2 _lut. (pmol/l)	SHBG foll. (nmol/l)	SHBG lut. (nmol/l)
**n**	37	37	37	34	35	35	35	29	29

**mean/SD**	34 ± 10	26.5 ± 5	12.6 ± 1.5	2.1 ± 1.5	15.0 ± 17.8	394.2 ± 317	355.5 ± 302	49.2 ± 22.2	49.6 ± 8.7

## Discussions

To our knowledge, this is the first time that the pattern of gene expression for three important proteoglycans in the healthy human breast has been shown during the menstrual cycle. We found that the gene expression for syndecan-1, syndecan-4 and decorin was significantly lower in the luteal phase than in the follicular phase of the menstrual cycle in parous women. Identification of the proteins was confirmed by immunhistochemistry, although significant correlations between the two menstrual phases were not obtained with this methodology.

Earlier studies of the healthy human breast during the menstrual cycle have reported various histologic changes in the epithelium and stroma [14,15]. For example, Ferguson et al. [13] used the indirect immunofluorescence to describe a pattern of increased staining of the ECM in weeks 1 and 4 of the menstrual cycle and reduced staining in weeks 2 and 3, while collagens remained unchanged. Stoeckelhuber et al. used an ultrastructural study to show a different organization between the proteoglycan-collagen associations in the luteal phase; this was explained by higher water content in the breast at this time [16]. The increase in distance was more prominent in the intralobular tissue than in the interlobular stroma. The importance of the interactions and co-dependencies between the epithelial cells and the ECM in breast tissue has been highlighted in the regulation of tumor progression [17]. Most of the gene expression required for transformation of normal tissue to a cancer *in situ *occurs early in the stroma and the epithelium, where stroma and epithelium cooperates and for acquisition of the invasive phenotype, the stroma is dominant over the epithelium[18]. Stromal changes have been documented in both pre-invasive breast lesions and tumors [19]. The SLRP decorin is found abundantly in the ECM. Decorin has been shown to inhibit the growth of both primary breast cancer xenografts and metastatic spread by inactivating the oncogenic ErbB2 protein in breast carcinoma cells [10] and downregulating members of the ERb tyrosine kinase family, leading to growth inhibition [10]. Previous studies have reported downregulation of decorin in breast cancer tissue [2], a situation which has been associated with a poor prognosis [9]. In a study on a rodent mammary carcinoma, Goldoni et al. described marked reduction in both primary tumor growth and metastatic spread after decorin protein was injected [20].

The physiologic reduction of gene expression in the luteal phase of the menstrual cycle may be a consequence of the effect of ovarian hormones [13]. Other authors have speculated on the gonoidal hormones effect on the ECM [21]. Progesterone, elevated during the luteal phase, interacts with growth factors (for example, HGF, EGF and IGF-1) and gives rise to both synergistic and inhibitory effects, influenced by the composition of the ECM [21]. The addition of progestin to estrogen hormonal replacement therapy increases the risk of cancer in the breast, while the opposite effect is well known in the uterine endometrium. In oophorectomized rats, the total levels of glycosaminoglycans decreased but, on the administration of estradiol, the levels of some HSPGs were increased. Sunil et al. found that progesterone administration, either alone or in combination with estradiol increased the levels of some proteoglycans in the rat mammary gland[22]. The syndecans, which are HSPGs can be either up-regulated or downregulated during the progression of a malignancy. Both syndecan-1 and syndecan-4 are overexpressed in an estrogen receptor -negative, highly proliferative breast carcinoma subtype [8]. Syndecan-1 can be redistributed from the epithelial cells to the stroma compartment in postmenopausal cancers [12,23,24]. In fact, increased expression of syndecan-1 has been associated with increased mammographic density as well as re-distribution from the epithelium to the stroma among postmenopausal women [11].

In our study of healthy breast tissue among premenopausal women, a decrease in gene signaling for syndecan-1 and -4 was observed in the luteal phase. Germeyer et al. examined syndecans-1 and -4 in the endometrium of the uterus and found a reciprocal expression with upregulation in the luteal phase of the menstrual cycle [25]. This was confirmed by Lai et al., who found that the expression of syndecan-1 was increased in the mid-luteal phase in uterine epithelial cells but was downregulated in the stroma during the early to late luteal phase, coinciding endometrial remodeling of the uterus [26]. From our results we can only conclude that decorin, syndecan-1 and syndecan-4 are regulated during the menstrual cycle in human breast tissue, providing an indication for further studies to clarify the influence of exogenous hormones.

## Conclusions

This study reveals the possible regulation of the proteoglycans syndecan-1, syndecan-4 and decorin during the menstrual cycle.

## List of abbreviations

ECM: extracellular matrix; RT-PCR: Reverse-transcription polymerase chain reaction; SHBG: Sex hormone binding globulin; EGF: epiderrmal growth factor; IGF-1: insulin growth factor; HSPG: heparane sulphate proteoglycan; SLRP: small leucine-rich proteoglycan.

## Competing interests

The authors declare that they have no competing interests.

## Authors' contributions

The study was planned by all authors. GH collected the data. EA and GH performed all the laboratory work and statistical analysis. GH wrote the manuscript with participation from GEO and EA. TN, EA and GEO reviewed the manuscript. All authors read and approved the final manuscript.
